# *N*‑Myristoyltransferase Inhibitors as a Potential Starting Point for the Development of Antischistosomal Agents

**DOI:** 10.1021/acsmedchemlett.6c00145

**Published:** 2026-03-30

**Authors:** Mareike Riedel, Collin Zimmer, Cécile Häberli, Jennifer Keiser, Christian Kersten

**Affiliations:** ± Institute of Pharmaceutical and Biomedical Science, 9182 Johannes Gutenberg University, Staudinger Weg 5, 55128 Mainz, Germany; ∇ Institute for Quantitative and Computational Biosciences, Johannes Gutenberg-University, BioZentrum I, Hanns-Dieter-Hüsch Weg 15, 55128 Mainz, Germany; # Department of Medical Parasitology and Infection Biology, 30247 Swiss Tropical and Public Health Institute, Kreuzstr. 2, 4123 Allschwil, Switzerland

**Keywords:** Schistosomiasis, N-Myristoyltransferase (NMT), NMT inhibitors, antischistosomal compounds, *Schistosoma mansoni*

## Abstract

Schistosomiasis remains a major global health challenge, and the threat of emerging praziquantel resistance highlights the need for new therapeutic strategies. Here, the *Schistosoma mansoni N*-myristoyltransferase was evaluated as a potential drug target. Six reported NMT inhibitors were tested for *Sm*NMT inhibition and binding, with two compounds showing low nanomolar potency. These also inhibited NMT of *S. hematobium* and *S. japonicum*, and exhibit favorable drug-like physicochemical properties. In addition, antiparasitic activity against newly transformed *schistosomula* and adult worms was investigated. The results confirm NMT as a viable target for drug discovery against schistosomiasis and provide a basis for further optimizations of *Sm*NMT inhibitors.

## Graphical abstract

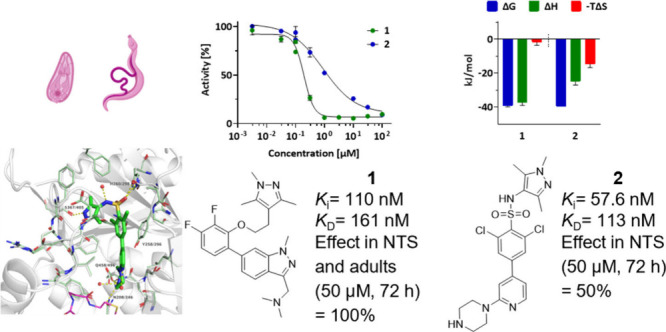

Schistosomiasis is an acute and chronic disease caused by parasitic worms of the genus *Schistosoma*.^1^ According to a WHO report on the number of schistosomiasis treatments in 2023, there were over 250 million treatments of preventive chemotherapy to treat schistosomiasis.^2^ In general, there are 19 known species of the genus *Schistosoma*, of which five species are pathologically relevant for humans.^3^ The disease is particularly prevalent in tropical and subtropical regions. The species *S. mansoni* is mostly distributed in Africa, the Middle East and South America.^4^

The parasite is usually transmitted through contact with fresh water containing human excrement with parasite eggs. The lifecycle of schistosomes is divided into one part that takes place in human and a part that takes place in the intermediate host (): First, the *cercariae* actively penetrates through the skin of the human host (1). They then lose their forked tails and develop into *schistosomula* (2). These enter the liver via the venous circulation, where they grow into adult worms (3). The adult worms leave the liver, copulate and migrate into the mesenteric veins of the intestine or rectum. There they lay their eggs, which are later excreted in the stool (4). The eggs hatch in water and release *miracidia* (5), which actively penetrate intermediate hosts such as snails. In the snail they develop into *sporocysts* (6), from which infectious *cercariae* are finally released (7).^5^ Since schistosomes pass through several distinct lifecycle stages, it is important for drug development to target the two human stages *schistosomula* and adult worms.^6^

The first treatment approaches against schistosomiasis date back to the early 20th century when different active substances against schistosomes were developed.^7^ With the establishment of suitable screening models, Oxamniquine (OAQ, ) was discovered in 1969.^8^ In 1972, a collaboration between Bayer AG and Merck KGaA led to the development of Praziquantel (PZQ).^9^ Today, PZQ is used in mass treatment programs for millions of people every year.^10^ It is the only drug currently recommended in the WHO control program and is also applied in a preventive manner.^11^

As PZQ is used worldwide due to its safety and efficacy against all relevant forms of schistosomiasis, concerns about the possible development of resistance are becoming increasingly important.^6^ Resistance to the drug OAQ, which was earlier used to treat schistosomiasis, has already been observed in *Schistosoma mansoni* both in laboratory studies and in the field.^12^ As a result, it has been proposed to develop derivatives of OAQ that are also effective but can overcome existing resistance.^13^ However, the main risk is that various studies have also shown that *S. mansoni* can develop resistance to PZQ in infected mice.^14^ In addition, there are reports of decreased cure rates and reduced sensitivity of schistosomes to PZQ in endemic areas, which increases the risk that resistance to the current standard medication against schistosomiasis will continue to spread.^15^ The development of new antischistosomiasis agents is urgently needed, partly due to the potential development of resistances.^16^ Various strategies can be considered: (1) the synthesis of PZQ analogs, (2) the development of new pharmacophores or (3) the identification of new targets.

In our investigation, we focused on approach (3), proposing the *N*-myristoyltransferase (NMT) as a potential new target for drug development against schistosomiasis. NMT catalyzes the transfer of the saturated fatty acid myristate from myristoyl-coenzyme A (MyrCoA) to the *N*-terminal glycine residue of substrate proteins.^17^ This myristoylation can occur both cotranslationally and post-translationally in eukaryotes and was shown to be crucial for signaling, protein transport and other cellular functions for different organisms.^18,19^ In the first step of the catalysis mechanism, MyrCoA binds to NMT, which triggers a structural change in the protein and opens the binding site for the substrate peptides.^20^ Subsequently, the substrate’s *N*-terminal glycine residue is deprotonated, allowing the myristoyl group to be transferred to the *N*-terminus of the protein. Finally, the free coenzyme A (CoA) and the *N*-myristoylated substrate protein are released.^21^

Using the traffic-light system of molecular target assessment for antiparasitic drug discovery^22^ criteria such as druggability, assay feasibility, structural information, and additional parameters that are critical for target assessment in drug discovery are well met for the *Schistosoma mansoni* NMT (*Sm*NMT). It can be considered a promising target, because NMTs were already investigated as a therapeutic target for human cancer^23^ and for various infectious diseases caused by viruses and fungi.^21,24^ Further, the enzyme is essential for the survival and spread of numerous parasitic pathogens, including *Trypanosoma*,^25,26^ *Leishmania* ^18,27^ and *Plasmodia spp.*.^27,28^ The NMT of *Trypanosoma brucei* has been successfully validated as a drug target with inhibitors curing mice from Human African trypanosomiasis (HAT).^29^ In addition, various selective and nonselective NMT inhibitors have also been tested against common cold viruses^30^ and parasitic^29,31^ infections, proving the druggability of NMT. For *Schistosoma* species, it was shown that NMT knockout results in embryotic lethality and sterility in adult worms.^32^ Even though there is no structure of *Sm*NMT available in the protein databank (PDB),^27^ the high sequence identity to the human NMT1 (*Hs*NMT1, overall sequence identity 54%, sequence similarity 69%) allows for homology modeling and structure-based approaches. While this high sequence identity makes selectivity a challenging task, it is worth mentioning that even nonselective NMT inhibitors were successful in curing mice from HAT.^29^ Further, *Hs*NMT inhibitors were also considered as treatment of the common cold hinting toward human NMT inhibitors being well tolerated in cell.^30,33^ Moreover, in a phase 1 study, a *pan*-NMT inhibitor was investigated for cancer therapy.^33^ Another important criterion in target assessment is assay feasibility, which was demonstrated by the availability of an NMT fluorescence-based assay in well-plate format.^24^ In addition, systematic evaluation of potential resistance mechanisms will be crucial to fully assess the suitability of this target for therapeutic intervention.

In this work, *Sm*NMT was investigated as a potential target for the treatment of schistosomiasis. For this purpose, *in vitro* inhibition studies were performed with previously reported NMT inhibitors (Figure 1). In addition, selected inhibitors were tested by isothermal titration calorimetry (ITC) for binding affinity, and against newly transformed *schistosomula* (NTS) and adult stages of *S. mansoni* to investigate their efficacy against the parasite. Reported chemotypes of NMT inhibitors include phenylindazole (**1**),^34^ pyrazole sulfonamides (**2–5**)^35^ and piperidinylindoles (**6**)^36,37^ as well as peptidic, peptidomimetic and other inhibitors, which are not part of this study.^37,38^

**Figure 1:**
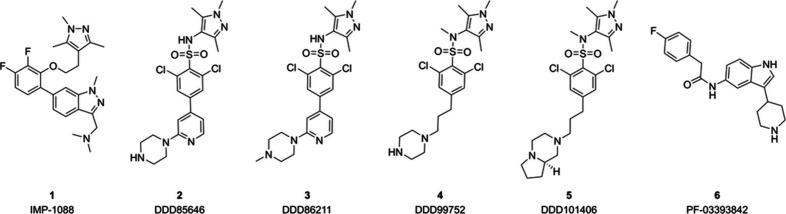
Reported NMT inhibitors under investigation.

Inhibitor **1** has been shown to inhibit human NMT1/2 with subnanomolar potency.^30^ Compounds **2–5** were described to inhibit NMTs from parasites, including *Trypanosoma*,^29^ *Plasmodia*,^39^ and *Leishmania spp*.^35,40^ Additionally, Pfizer identified inhibitor **6**, which has also been tested for its ability to inhibit parasitic NMT.^41^ The binding modes of multiple compounds have been described using X-ray crystallography with human or parasitic NMTs.^35,36^ The highly similar binding site between *Sm*NMT and *Hs*NMT1 (Figure 2) leads to the hypothesis that binding modes and potency might be conserved between the two enzymes.^36,42^ A common feature among reported NMT inhibitors is a basic center mimicking the substrates *N*-terminal Gly residue, which interacts with the NMT *C*-terminus in the active site via ionic interactions (Gln496 in *Hs*NMT1). Further, most reported inhibitors interact with Ser405. These two features are connected by a hydrophobic, usually aromatic linker. Depending on the type of inhibitor, Tyr296 can adopt different conformations often contributing to selectivity.^34,36^

**Figure 2:**
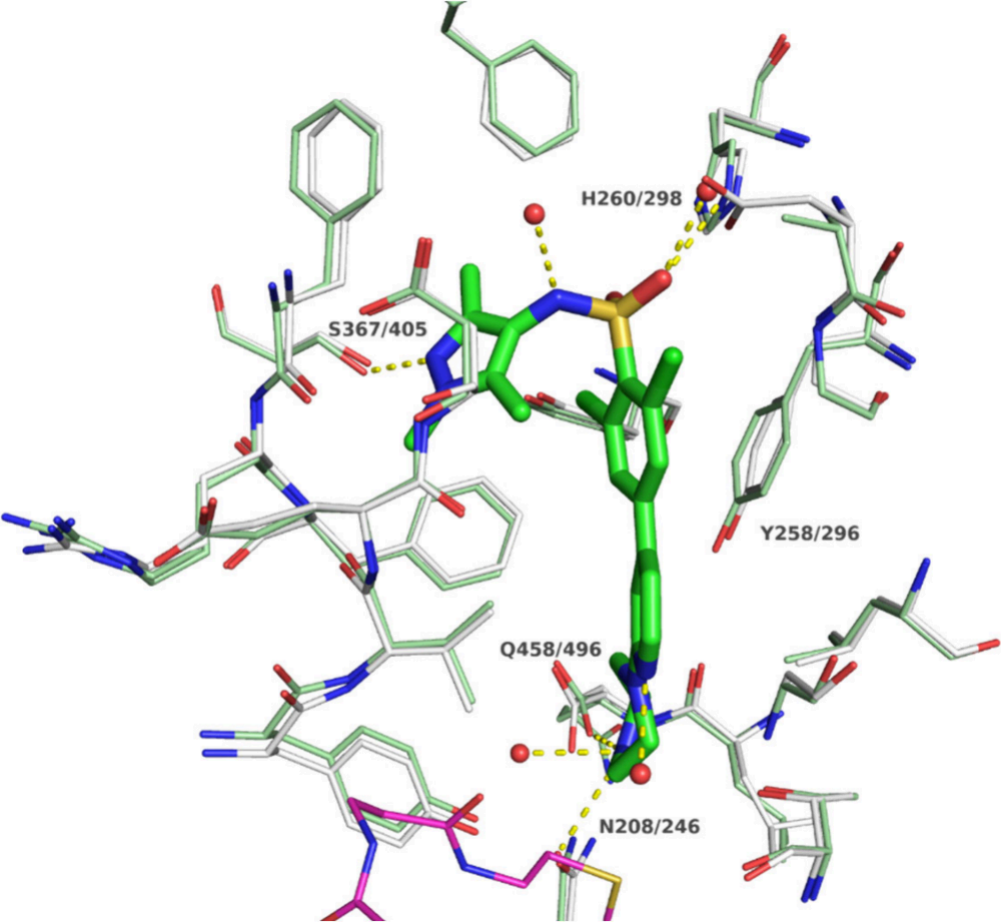
Binding site view of superposed of *Sm*NMT AlphaFold3 model^43^ (white carbon atoms and transparent cartoon) and *Hs*NMT1 in complex with **2** (PDB-ID: 3IWE, green carbon atoms). For clear view, only key residues interacting with the ligand are labeled with IDs (*Sm*NMT/*Hs*NMT1). Part of the cofactor MyrCoA is shown with magenta-colored carbon atoms for orientation.

Inhibitors **1–6** were characterized against various NMTs previously. For the investigation of the six inhibitors, the *Sm*NMT (residues 74–458) with an *N*-terminal hexahistidine-tag was expressed in *E. coli*. Their inhibition constants (*K*_i_) for *Sm*NMT were determined using a fluorescence-based assay (Table 1, ).^24^ For the measurement of the enzyme activity the polypeptide GSNKSKPK-amide was used. The formation of free CoA during the enzymatic reaction was detected in a coupled reaction with the fluorogenic dye 7-diethylamino-3-(4-maleimidophenyl)-4-methylcoumarin (CPM). Inhibitors **1** and **2** showed the highest potency, with *K*_*i*_ values of 110 and 57.7 nM, respectively. Inhibitors **3–5** were less potent with *K*_*i*_ values in the three-digit nanomolar range. For **6**, no *K*_*i*_-value could be unambiguously determined due to limited solubility above 500 μM under assay conditions. Notably, despite the high similarity between *Sm*NMT and *Hs*NMT1 (Figure 2), inhibition of the human enzyme was generally higher hinting toward the requirement of improved selectivity for optimization. Only inhibitor **2** shows similar inhibition of both enzymes.

**Table 1:** Inhibition Constants (*K*_i_) of Inhibitors **1**–**6** against *Sm*NMT and Reported *Hs*NMT1 Inhibition^a^

cpd	*K*_i_ [nM] *Sm*NMT	*K*_i_ [nM] *Sh*NMT	*K*_i_ [nM] *Sj*NMT	*K*_i_ [nM] *Hs*NMT1
**1**	110 ± 6	62.3 ± 8.7	64.5 ± 3.0	IC_50_ < 1 nM, *K*_D_ ≤ 210 pM^30^
**2**	57.7 ± 9.4	132 ± 15	72.6 ± 8.7	31.6 ^35^
**3**	293 ± 19	n.d.	n.d.	13.3 ^35^
**4**	718 ± 113	n.d.	n.d.	96.4 ^35^
**5**	837 ± 68	n.d.	n.d.	430 ^35^
**6**	>28,300	n.d.	n.d.	73,200 ^35^

a For inhibitors **1** and **2**, *K*i values were additionally determined for *Sh*NMT and *Sj*NMT. *K*_i_-values were calculated from *K*_M_ and IC_50_-values () using the Cheng-Prusoff equation.^44^

For the most potent inhibitors **1** and **2**, ITC was performed as an orthogonal binding assay (Figure 3, ). The obtained *K*_D_ values are *K*_D_ *=* 161 ± 9.3 nM for **1** and *K*_D_ *=* 113 ± 1.4 nM for **2**, and slightly higher than the determined *K*_i_ values, but within the expected range when comparing different methods. The comparison of the thermodynamic binding profiles shows that binding enthalpy Δ*H*° is lower for **2** compared to **1**. However, binding entropy -TΔS° is higher for compound **2**, resulting in similar Δ*G*° values.

**Figure 3:**
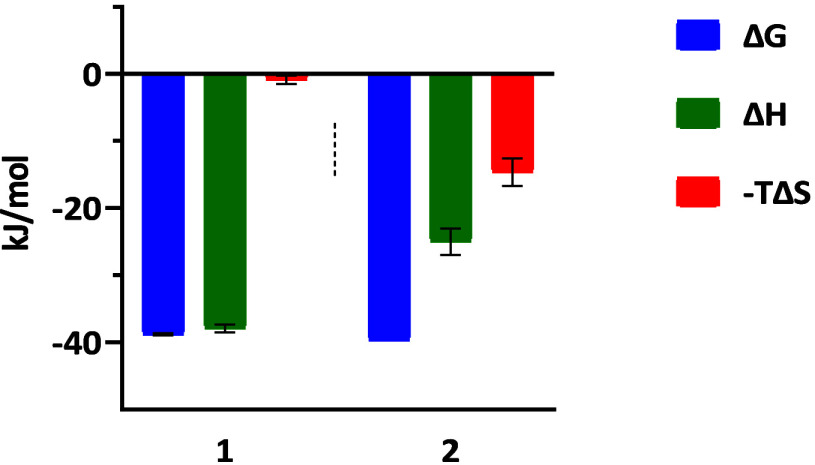
Thermodynamic profiles of compounds **1** and **2** binding to *Sm*NMT. **1**: Δ*G*° = −38.8 ± 0.2 kJ/mol, Δ*H*° = −37.9 ± 0.6 kJ/mol, −*T*Δ*S*° = −0.9 ± 0.6 kJ/mol and *K*_D_ = 161 ± 9 nM, **2**: Δ*G*° = – 39.7 ± 0.0 kJ/mol, Δ*H*° = −25.0 ± 2.0 kJ/mol, −*T*Δ*S*° = 14.7 ± 2.1 kJ/mol and *K*_D_ = 113 ± 1 nM.

To assess inhibitory activity of the compounds against NMTs from other *Schistosoma* species, the two most potent inhibitors **1** and **2** were also tested against *Sh*NMT and *Sj*NMT which share a high sequence identity with *Sm*NMT (Table 1, ). For *Sh*NMT, *K*_i_ values of 62.3 nM for inhibitor **1** and 132 nM for inhibitor **2** were determined. For *Sj*NMT, *K*_i_ values of 64.5 nM for inhibitor **1** and 72.6 nM for inhibitor **2** were obtained. Both compounds thus also show similar inhibitory activity in the low nanomolar range against the NMT homologues of multiple *Schistosoma* species. These results suggest that the investigated inhibitors represent a promising starting point for the development of active substances against NMTs of several human-pathogenic *Schistosoma* species.

Despite target inhibition, entering the parasite is crucial for drugs to be effective against *Schistosoma spp*. Physicochemical properties of 57 FDA-approved antischistosomiasis drugs, hits and derivatives were recently investigated.^45^ The molecular weight of all compounds was below 500 g/mol. The topological polar surface area (TPSA) values showed a wide range from 7–125 Å^2^ due to the high structural diversity of the investigated compounds. It is noteworthy that more than 65% of the antischistosomal substances had logD_7.4_ values above 3, although a range of 1–3 is usually recommended for good oral bioavailability.^46^ This suggests that higher lipophilicity may be required for effective control of schistosomes, although this may be at the expense of oral bioavailability. These and additional common rule-of-5 parameters, H-bond donors (HBD) and acceptors (HBA), number of rotatable bonds, acidic and basic centers – especially the latter due to the requirement of a basic center in most reported NMT inhibitors – were calculated. Further, the number of molecules was expanded by substances with reported activity against *S. mansoni schistosomula* or adult worms from ChEMBL.^47^ Parameters were compared with FDA-approved drugs and the NMT inhibitors under elucidation as well as current therapeutics PZQ and OAQ (Table 2). Compared to FDA-approved drugs, compounds with antischistosomal activity tend to lack acidic and basic centers and be overall less polar with lower numbers of HBD and HBA resulting in a lower TPSA and higher logP and logD_7_ values in line with previous findings.^45^ However, the presence of a basic amine in OAQ indicates that positive charges do not need to be generally avoided. Further, structural complexity is slightly reduced with fewer chiral centers and rotatable bonds. The comparison of NMT inhibitors **1–6** with the reference drugs shows that for the numbers of HBA and HBD, some variation seems to be tolerated. While PZQ contains no HBD, OAQ has four. The NMT inhibitors tested are within this range. In terms of HBA, **1–6** contain between four and eight acceptor groups, whereby PZQ has four HBA and OAQ has six HBA. The molecular weight of most compounds is below the threshold value of 500 g/mol specified in the guidelines for drug-likeness.^48^ Only compounds **3** and **5** slightly exceed this value. Antischistosomal compounds including PZQ and OAQ tend to be smaller with around 300 g/mol. TPSA values of the tested inhibitors are between 40 and 100 Å^2^, which is within the favorable range for oral bioavailability^46^ and similar to PZQ (41 Å^2^) and OAQ (95 Å^2^). Finally, regarding lipophilicity, four of the six inhibitors (**2** – **5**) exhibit logP values between 1 and 3, considered the optimal range for oral bioavailability, or higher in terms of antischistosomal compounds. **1, 3** and **6** are slightly more lipophilic with logP of 4.0, 3.0 and 3.8, respectively, but such an increased lipophilicity is a tolerated if not favorable feature for antischistosomal drugs.^45^

**Table 2:** Physicochemical Properties (Calculated with MOE,^49^ p*K*_a_ Values using MarvinSketch^50^) of FDA-Approved Drugs, Compounds with Antischisosomal Activity (pChEMBL-value >4 Corresonding to IC_50_ or EC_50_ Values below 100 μM against *Schistosomula* or Adult Worms of Different Ages), NMT-Inhibitors **1**–**5** and Approved Antischistosoma Drugs PZQ and OAQ^a^

parameter	FDA (*n* = 1251)	Anti-*S. mansoni* (*n* = 167)	1	2	3	4	5	6	PZQ	OAQ
MW[g/mol]	376.5	313.4	453.5	495.4	509.5	474.5	514.5	351.2	312.4	279.3
acidic centers (p*K*_a_)	0.79	0.14	0	1 (6.6)	1 (6.5)	0	0	0	0	0
basic centers (p*K*_a_)	0.58	0.05	1 (7.5)	1 (8.8)	1 (7.7.)	1 (9.3)	1 (9.4)	1 (10.0)	0	1 (9.9)
chiral centers	2.3	1.0	0	0	0	0	1	0	1	1
HBD	2.6	0.6	1	3	2	2	1	3	0	4
HBA	6.3	4.7	6	8	8	7	7	4	4	6
rotatable bonds	6.9	5.0	7	6	6	8	7	6	3	6
TPSA [Å^2^]	92	55	49	97	85	75	63	57	41	95
logP	2.3	3.9	4.0	2.7	3.0	1.9	2.8	3.8	2.2	1.9
logD_7_	1.0	2.7	3.5	3.0	3.3	1.6	2.0	0.4	3.2	–0.7

a Average values are provided for FDA-approved drugs and anti-S. mansoni compounds from ChEMBL.

Lastly, inhibitors **1–5** were tested against newly transformed *schistosomula* (NTS) at concentrations of 50 μM and 10 μM. In this assay, compound **1** resulted in death of all *schistosomula* at the 50 μM concentration, while **2–5** showed 50% or less activity. At a lower concentration of 10 μM, all NMT inhibitors showed activity between 26 and 43%. Based on these results, compound **1**, one of the strongest *Sm*NMT inhibitors (Table 1) with good physicochemical properties (Table 2) and the strongest NTS effect at the 50 μM concentration (Table 3), was further tested on adult *S. mansoni* at 50 and 10 μM. Remarkably, it also achieved 100% lethality in adult parasites at 50 μM and showed an activity of 48% at 10 μM concentration. The higher concentrations required for antischistosomal activity compared to NMT inhibition, indicates limited permeability of the compounds. This is in line with the described physicochemical properties (Table 2) including the higher potency of compound **1** which is smaller, less polar and less basic compared to **2** – **5**. To exclude that the antischistosomal effect is caused by general cytotoxicity at these high concentrations, the two most active compounds **1** and **2** were tested against HEK293 cells (). At a concentration of 10 μM, no cytotoxicity in HEK293 cells was observed. At 50 μM, where inhibitor **1** caused complete death of NTS and adult worms, moderate cytotoxicity was detected in HEK293 cells, with more than 50% of cells surviving after 72 h of treatment with compounds **1** or **2**. This shows that the lethal effect is stronger in schistosomes than the general cytotoxicity in HEK293 cells, especially in the case of compound **1**.

**Table 3:** In Vitro Effects of **1**– **5** on *S. mansoni* NTS and Adult Worms^a^

	NTS		adult	
cpd	Effect in% (±SD) (72 h), 50 μM	Effect in% (±SD) (72h), 10 μM	Effect in% (72h) (±SD), 50 μM	Effect in% (72h) (±SD), 10 μM
**1**	100 ± 0	26 ± 4	100 ± 0	48 ± 3
**2**	50 ± 4	35 ± 2	n.d.	n.d.
**3**	44 ± 8	43 ± 2	n.d.	n.d.
**4**	44 ± 2	33 ± 2	n.d.	n.d.
**5**	44 ± 0	35 ± 2	n.d.	n.d.

a SD: standard deviation, n.d.: not determined.

In summary, NMT of *Schistosoma spp*. can be considered a potential drug target. The results indicate that reported NMT inhibitors are effective against *Sm*NMT, *Sh*NMT and *Sj*NMT reaching nanomolar potency (Table 1, ) and that the presence of a basic center in the compounds under investigation, which is required for interaction with NMT, appears to be tolerated in *S. mansoni* permeation (as also seen in OAQ, Table 2, ). Room for improvement can be found in selectivity over human NMT and antischistosomal activity, the latter probably limited by permeation.

## Supplementary Material





## Notes

The Supporting Information is available free of charge on the ACS Publications Web site. Material and Methods, Figures S1–S14, chemical analytics (PDF)
Plasmid maps (DNA) (ZIP)

## Notes

Safety statement: No unexpected or unusually high safety hazards were encountered.

## Abbreviations

ChEMBL: Chemical Database of Bioactive Molecules
CoA: Coenzyme A
CPM: 7-diethylamino-3-(4-maleimidophenyl)-4-methylcoumarin
FDA: Food and Drug Administration
HAT: Human African Trypanosomiasis
HBA: Hydrogen Bond Acceptor
HBD: Hydrogen Bond Donor
HEK: Human Embryonic Kidney
*Hs*NMT1: Human *N*-Myristoyltransferase 1
ITC: Isothermal Titration Calorimetry
MOE: Molecular Operating Environment
MW: Molecular Weight
MyrCoA: Myristoyl-Coenzyme A
n.d.: not determined
NMT: *N*-Myristoyltransferase
NTS: Newly Transformed *Schistosomula*
OAQ: Oxamniquine
PDB: Protein Data Bank
PZQ: Praziquantel
*Sh*NMt: *Schistosoma hematobium N*-Myristoyltransferase
*Sj*NMT: *Schistosoma japonicum N*-Myristoyltransferase
*Sm*NMT: *Schistosoma mansoni N*-Myristoyltransferase
TPSA: Topological Polar Surface Area
