# Supplementary material for: N‑Myristoyltransferase Inhibitors as a Potential Starting Point for the Development of Antischistosomal Agents
Source: ACS Med Chem Lett. 2026 Mar 30;17(4):945–51. doi: 10.1021/acsmedchemlett.6c00145 (PMC13071649; doi:10.1021/acsmedchemlett.6c00145)
Supplement: Supplementary file 1 [file ml6c00145_si_001.pdf]

Supporting Information

N-Myristoyltransferase Inhibitors as a Potential Starting Point for the Development of Antischistosomal Agents

Mareike Riedel[±], Collin Zimmer[±], Cécile Häberli[#], Jennifer Keiser[#], Christian Kersten[±,∇*]

[±]Institute of Pharmaceutical and Biomedical Science, Johannes Gutenberg University, Staudinger Weg 5, 55128 Mainz, Germany.

[∇] Institute for Quantitative and Computational Biosciences, Johannes Gutenberg-University, BioZentrum I, Hanns-Dieter-Hüsch Weg 15, 55128 Mainz, Germany

[#]Department of Medical Parasitology and Infection Biology, Swiss Tropical and Public Health Institute, Kreuzstr. 2, 4123 Allschwil, Switzerland

*Corresponding author:
E-mail address: kerstec@uni-mainz.de

Content:

Material and methods 2
S. mansoni lifecycle and approved antischistosomal agents 4
Plasmid information and sequence identity 5
Michaelis-Menten kinetics 5
Inhibition data of inhibitors 1-6 6
Cytotoxicity 7
ITC results 7
LC/MS analytics 8

Material and methods

Chemistry. Compound **1** (Cay25366) was purchased from Biomol/Cayman Chemicals. Compound **2** (HY-103056) was purchased from MedChemExpress. The synthesis and chemical analysis of inhibitors **3**–**6** was described previously.[35] The purity of these compounds was re-determined by LC/MS analysis confirming identity and purity > 95%. (Figures S7–S12). The chromatograms and mass spectra were obtained by LC/MS using a Waters Alliance e2695 Separations Module with an analytical Pursuit XRs C18 (4.6 × 200 mm, 5.0 µm) column coupled to a Waters Acquity QDa single quadrupole detector. Compounds **1**–**3** and **5**–**6** were separated using a mobile phase of MeCN/H_2O + 0.1% HCOOH (gradient 10:90 → 100:0; flow rate 3.0 mL/min; t = 10 min), whereas for Compound **4** a mobile phase of MeCN/H_2O + 0.1% HCOOH (gradient 70:30 → 90:10; flow rate 1.2 mL/min; t = 10 min) was used. Mass spectra were recorded in the positive mode. The UV detection wavelengths were dependent on the compound absorbance at 210 nm or 254 nm.

Computational analysis. The *N*-terminally truncated protein sequences of *Hs*NMT1 (UniProt: P30419) and schistosomal NMTs (Uniprot: *Sm*NMT – A0A3Q0KKD2, *Sj*NMT – Q5DFY0, *Sh*NMT – A0A922LXN3) were used for the sequence alignment and identity/similarity calculation with MOE (Molecular Operating Environment, MOE 2024.0601; Chemical Computing Group ULC: 1010 Sherbooke St. West, Suite #910, Montreal, QC, Canada, H3A 2R7, 2024.)[49]. Based on the *Sm*NMT sequence (residues 74-458), a homology model was built using the AlphaFold3 webserver.[43] Physicochemical properties were calculated using MOE and pK_a-values were predicted with MarvinSketch (MarvinSketch 17.17.0, ChemAxon Ltd. 2017).[50] Figures were made with BioRender (https://www. biorender.com/) and PyMOL (Open-Source Build, Version 3.1.0a0, Schrödinger, LLC.).

Protein expression. The plasmid coding for *N*-terminal 6xHis-tagged schistosomal NMT wildtypes (residues 74–458 for *Sm*NMT, 79-463 for *Sj*NMT and 74-458 for *Sh*NMT, matching common *N*-terminal truncation of parasitic and human constructs) were designed according to UniProt-IDs: A0A3Q0KKD2 , Q5DFY0 and A0A922LXN3, respectively in a pet28a(+)TEV vector and purchased from GenScript, Piscataway Township, USA. NMTs were expressed in *E. coli* BL21 cells using LB medium supplemented with 0.04 mM kanamycin. Cultures were incubated at 37 °C and 200 rpm until an OD_{600} of 0.6–0.8 was reached. Protein expression was induced by the addition of 0.5 mM IPTG (Thermo Scientific, 10849040), followed by incubation at 16 °C for 16 hours. Cells were harvested by centrifugation at 10.000×g for 15 minutes at 4 °C, then resuspended in lysis buffer (50 mM HEPES at pH 7.5, 500 mM NaCl, 5% glycerol, and 20 mM imidazole). Cell lysis was achieved by treatment with lysozyme (Carl Roth, 8259.2), DNase I (NEB, M0570S), and a protease inhibitor cocktail tablet (Merck, 11836170001), followed by sonication (10 cycles of 30 seconds with 30 seconds intervals on ice). Cell debris was removed by centrifugation at 15.000×g for 40 minutes at 4 °C. The clarified lysate was loaded onto a 5 mL HisTrap column, and the bound protein was eluted using a linear imidazole gradient from 20 mM to 200 mM. Fractions containing NMT were pooled and concentrated using Amicon® Ultra Centrifugal Filters, 10 kDa molecular weight cutoff (MWCO) from Sigma Aldrich. Protein identity and purity were confirmed by SDS–PAGE analysis with Coomassie blue staining.

Enzymatic assay. Enzyme activity and inhibition were measured using a fluorescence-based assay as described for other NMTs,[24] performed on a Tecan M200 Infinite Pro or Tecan Spark plate reader with excitation at 380 nm and emission at 470 nm. Assays were conducted in 110 µL with sodium phosphate buffer (pH 7.8) containing 0.5 mM EDTA and 100 nM *Sm*NMT, *Sh*NMT or *Sj*NMT. The peptide GSNKSKPK-amidation (Selleck Chemicals LLC) was used as substrate, and MyrCoA (Cay39777-5, Biomol) served as the cofactor. Free CoA generated during the enzymatic reaction was detected using the thiol-reactive fluorescent dye 7-diethylamino-3-(4-maleimidophenyl)-4-methylcoumarin (CPM, Sigma Aldrich, 96669-10MG-F). For K_M determination, a continuous 45-minute assay was performed using a concentration of 100 nM NMT, 30 µM MyrCoA, 20 µM CPM

and varying peptide substrate concentrations (1–64 µM). The K_M value was calculated from the initial reaction rate over the first 15 minutes for *Sm*NMT and 10 minutes for *Sh*NMT and *Sj*NMT. For IC_{50} determination, the peptide substrate was used at 30 µM and MyrCoA at 4 µM, with 8 µM CPM. Inhibitors were tested over a concentration range of 1 nM to 100 µM in a 3.16-fold (half-logarithmic) dilution series, depending on the compound. The final DMSO concentration in all assays was 0.9%. All experiments were conducted at least in triplicates. Inhibition constants (K_i) were calculated from IC_{50} and K_M values using the Cheng–Prusoff equation.[44]

Isothermal titration calorimetry. Prior to ITC experiments, the buffer of the enzyme was exchanged to a 50 mM phosphate buffer with pH 7.8 using an Amicon® Ultra Centrifugal Filter, 10 kDa MWCO. Compounds **1** and **2** were diluted to a final concentration of 100 µM with ITC buffer containing 0.9% and 1.0% DMSO, respectively. *Sm*NMT was adjusted to a final concentration of 10 µM and matched DMSO content. A MicroCal PEAQ-ITC Automated (Malvern Panalytical) with a 190 µL Hastelloy cell and a 40 µL injection syringe was used for the ITC measurements. All experiments were performed in duplicate or triplicate at 25 °C, with a stirring speed of 750 rpm and a reference power of 42 µW. A total of 19 injections of 2 µL each were added into the reaction cell at an injection rate of 0.5 µL/s and an interval of 150 s. The data was analyzed using the MicroCal PEAQ-ITC Analysis Software (version 1.21).

***In vitro* testing on *Schistosoma mansoni* newly transformed schistosomula (NTS).** Harvested *S. mansoni* cercariae (Liberian strain) obtained from infected *Biomphalaria glabrata* snails were mechanically transformed into newly transformed schistosomula (NTS) following standard procedures. Briefly, snails were placed under light in the morning to stimulate cercarial shedding. The cercarial suspension was collected, cooled for 30 minutes and then vigorously pipetted (30X) and vortexed (3 minutes). The suspension was then placed in the incubator (37°C and 5% CO_2) for 30 minutes and the vortexing and pipetting steps were repeated. The tails were separated from the heads by rinsing three times with cold HBSS. NTS were then incubated overnight in culture medium and used the next day. 30-40 NTS were placed in each well of a 96-well plate with culture medium and all 5 compounds were tested at 50µM and 10µM for a final well volume of 200-250 µl. Culture medium was composed of M199 medium (Gibco, Waltham MA, USA) supplemented with 5% Horse serum (Gibco, Waltham MA, USA) 1% penicillin/streptomycin mixture (Invitrogen, 100 U/ml) and 1% Mäser Mix. Each compound was tested in triplicate and repeated once. NTS incubated with no more than 1% DMSO served as control. NTS were kept in the incubator at 37°C and 5% CO_2 for up to 72 hours. After 72 hours, the condition of the NTS was microscopically evaluated. Worms are scored as 0= dead; 0.25-1=reduced motility and significant tegument damage; 1.25-2= reduced motility or marked tegument damages 2.25-3= viable, nice tegument, good motility.

***In vitro* testing on *Schistosoma mansoni* adult worms.** All experiments were carried out in accordance with Swiss national and cantonal regulations on animal welfare under permission number 545. Female NMRI mice (age 3 weeks, weight ca. 14-20 g) were purchased from Charles River (Sulzfeld). The animals were allowed to adapt for one week under controlled conditions (20-23°C, 45-65% humidity, 12 hours light, and free access to water and rodent diet) before experimental handling. To obtain adult schistosomes, NMRI mice were infected subcutaneously with 80 to 100 cercariae. After 49 days, the mice were euthanized with CO_2 and the worms collected from the hepatic portal and mesenteric veins. 3 pairs of adult worms were placed in each well of a 24-well plate with 2-2.5 ml culture medium and one the active drug was tested at 50 µM and 10 µM). Culture medium was composed of RPMI 1640 (Invitrogen, Carlsbad, CA) supplemented with 5 % fetal calf serum (iFCS, 100 U/ml) and 1% penicillin/streptomycin mixture (Invitrogen, 100 U/ml). Each compound was initially tested in duplicate and repeated once. Schistosomes incubation with no more than 1% DMSO served as control. Worms were kept in an

incubator at 37°C and 5% CO_2 for up to 72 hours. After 72 hours, the condition of the worms was microscopically evaluated as described above for NTS.

Cell viability assay. The cell viability assay was conducted using HEK293 cells and the CellTiter-Glo® 2.0 Cell Viability Assay (Promega). Cells were cultured in DMEM (Dulbecco's Modified Eagle Medium GlutaMAX™ Supplement, pyruvate, 10% FBS, penicillin/streptomycin) at 37 °C in a humidified atmosphere containing 5% CO_2. A total of 2,000 cells were seeded per well in white 96-well half-area plates (Greiner Bio-One CELLSTAR). After overnight incubation, the cells were treated with the compounds at final concentrations of 10 µM and 50 µM. The final concentration of DMSO was 0.1%. After 72 h of incubation, the CellTiter-Glo assay was performed according to the manufacturer's protocol. The CellTiter-Glo reagent was added to each well and incubated at room temperature. Luminescence was measured using a Tecan Spark 10M plate reader. Cell viability of treated cells was calculated relative to the DMSO-treated control wells. Each condition was tested in five replicates.

S. mansoni lifecycle and approved antischistosomal agents

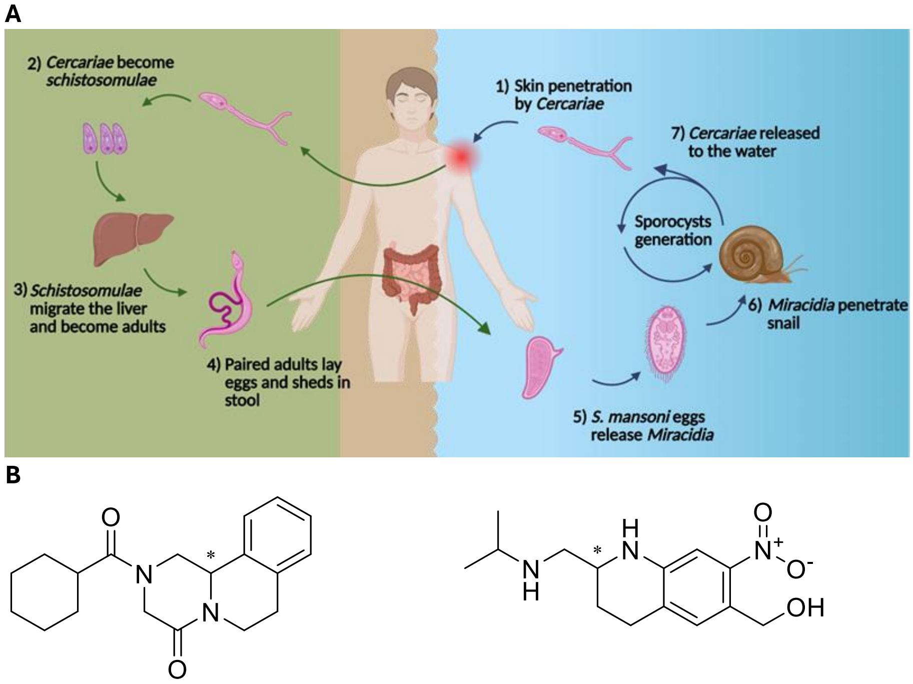

Figure S 1: A) Lifecycle of Schistosoma mansoni parasites inside its intermediate host and human based on reference 5. Created with BioRender.com. B) Molecular structure of antischistosomal agents PZQ (left) and OAQ (right).

Plasmid information and sequence identity

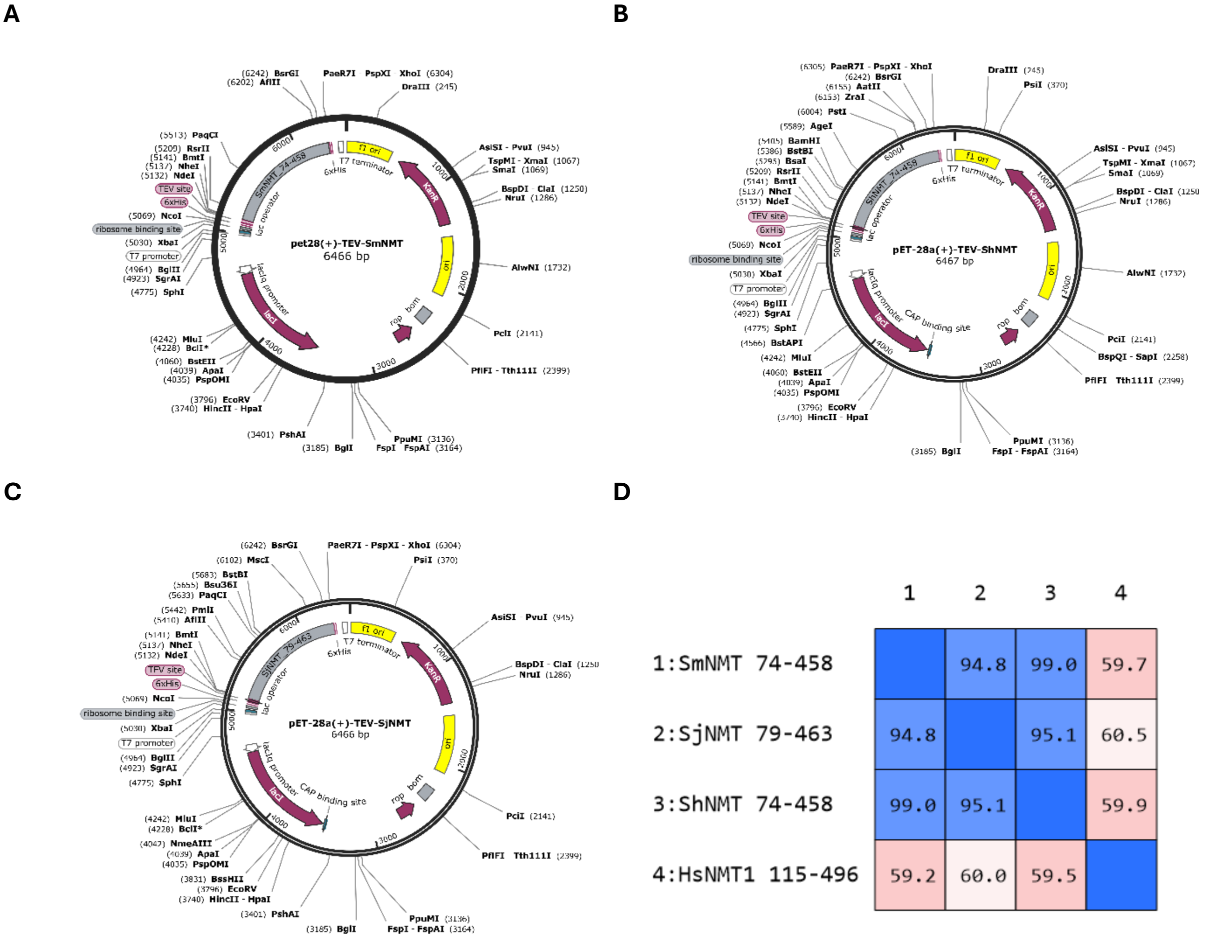

Figure S 2: Plasmid map of the A) pet28a(+)-6xHis-TEV-*Sm*NMT, B) pet28a(+)-6xHis-TEV-*Sh*NMT and C) pet28a(+)-6xHis-TEV-*Sj*NMT vector used for recombinant NMT expression in *E.coli*. The plasmid maps are also provided as *.dna file in the Supporting Information. D) Sequence identity between *schistosoma* NMTs under investigation and *Hs*NMT1. Calculated with MOE.

Michaelis-Menten kinetics

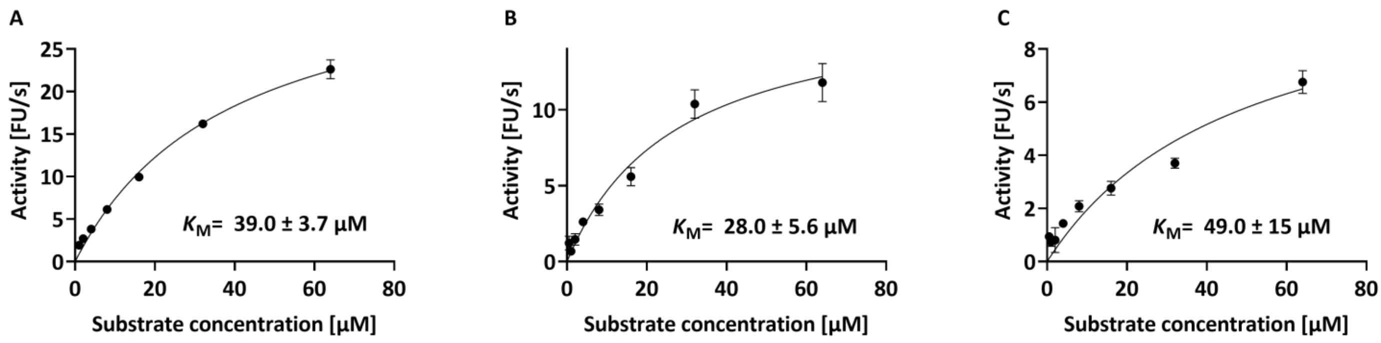

Figure S 3: Michaelis Menten kinetics of A) *Sm*NMT, B) *Sh*NMT and C) *Sj*NMT with substrate GSNKSKPK (pp60$^{src}_{(2-9)}$.)

Inhibition data of inhibitors 1-6

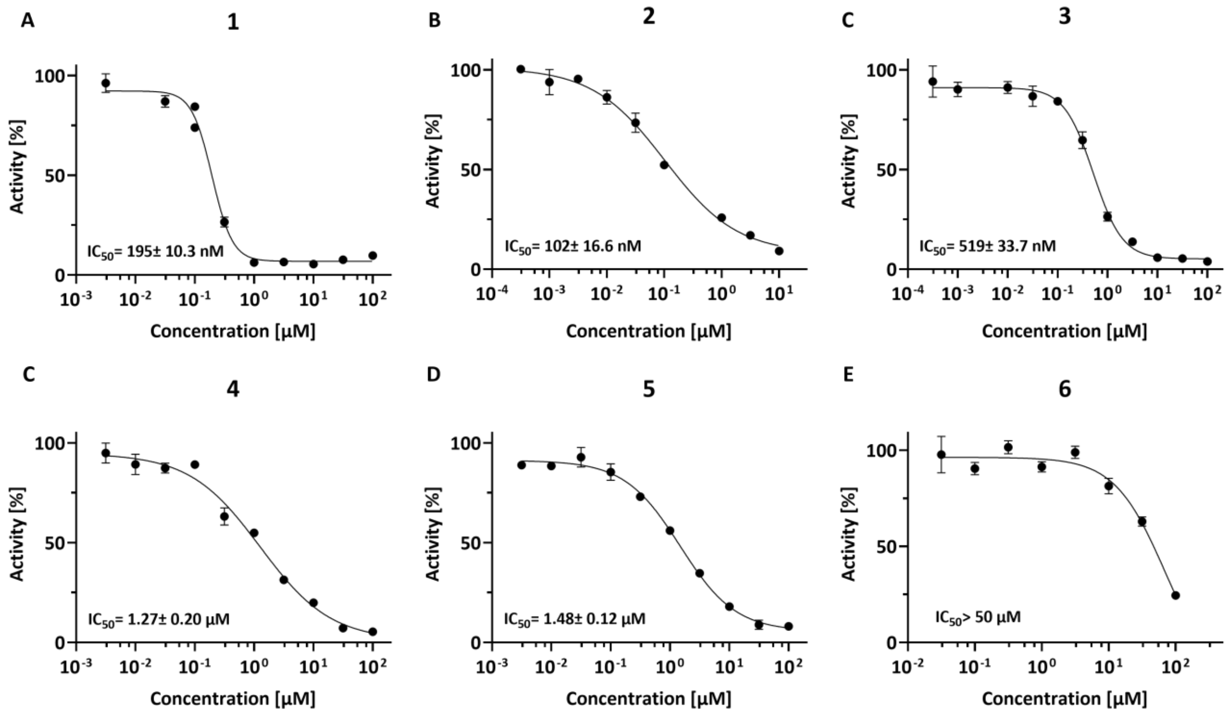

Figure S 4: IC_{50} curves of tested inhibitors against *Sm*NMT. A) **1**, B) **2**, C) **3**, D) **4**, E) **5**, F) **6**.

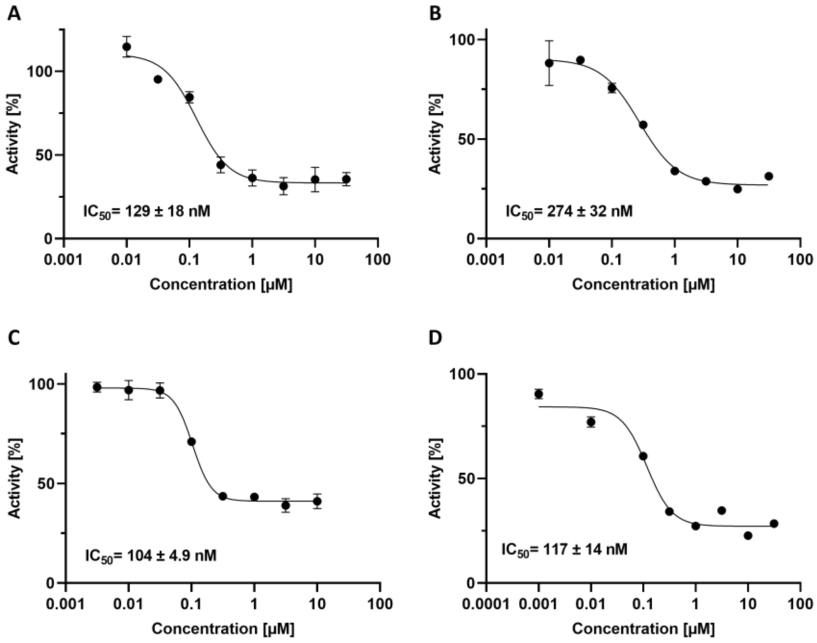

Figure S 5. IC_{50} curves of tested inhibitors against *Sh*NMT. A) **1**, B) **2** and *Sj*NMT C) **1**, B) **2**.

Cytotoxicity

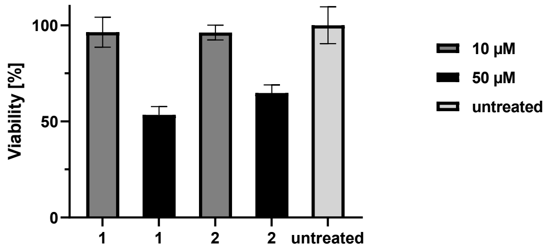

Figure S 6: Cell viability of HEK293 cells in presence of inhibitors **1** and **2** at final concentrations of 10 µM and 50 µM.

ITC results

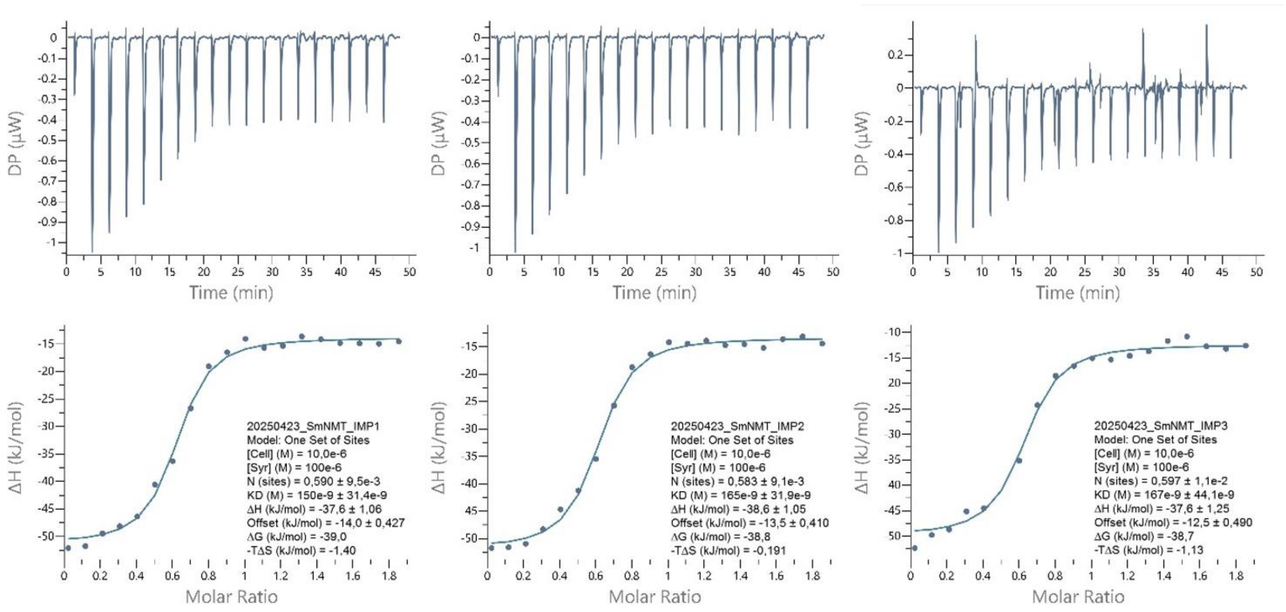

Figure S 7: ITC thermograms and binding isotherms of compound **1** binding to *Sm*NMT.

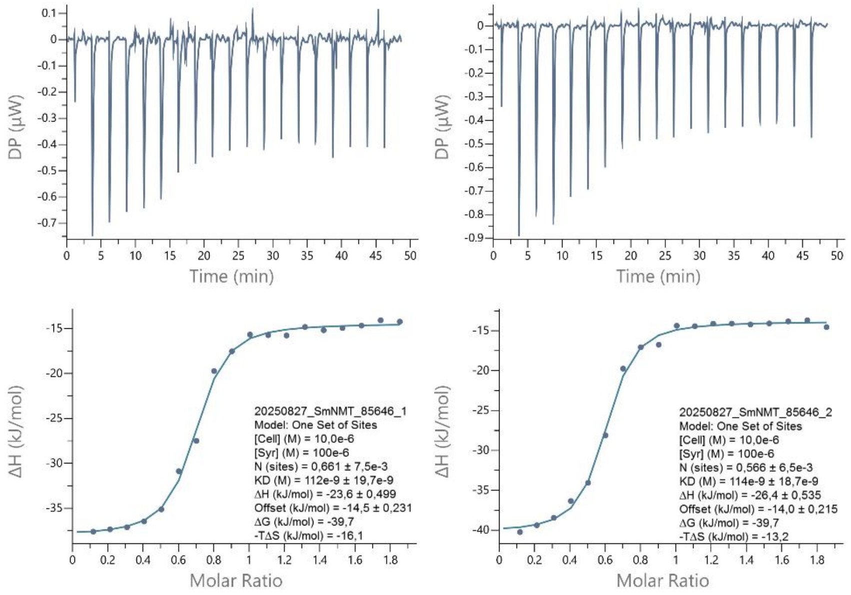

Figure S 8: ITC thermograms and binding isotherms of compound **2** binding to *Sm*NMT.

LC/MS analytics

1

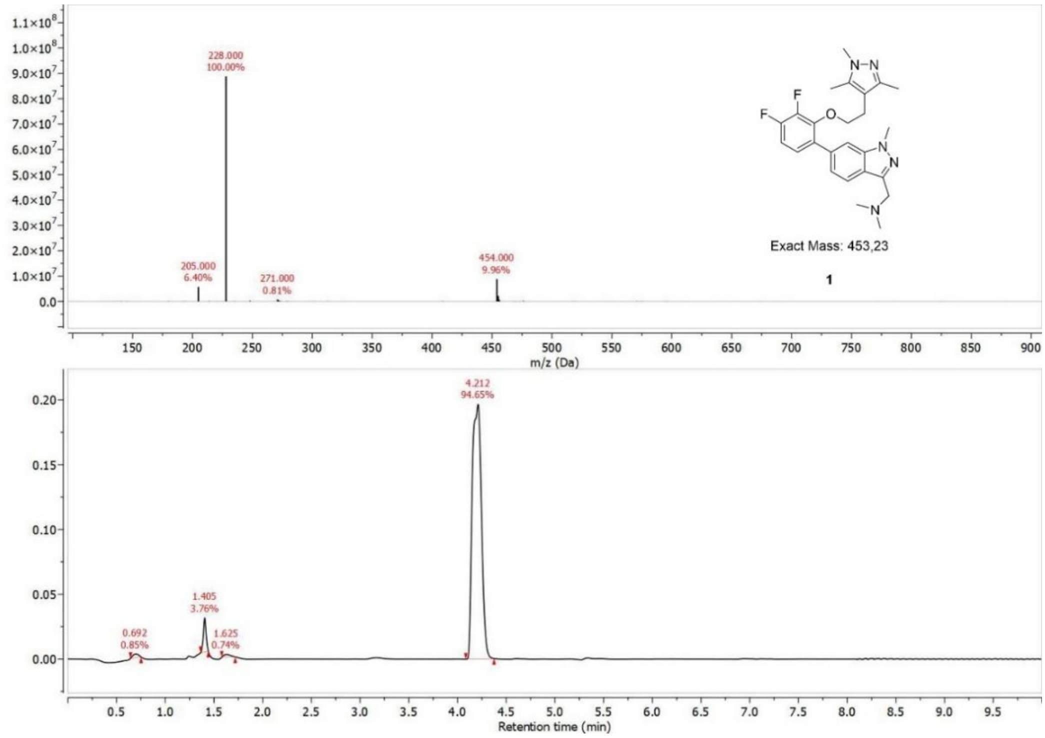

Figure S 9: Mass spectrum and UV chromatogram (254 nm) of compound **1.**

2

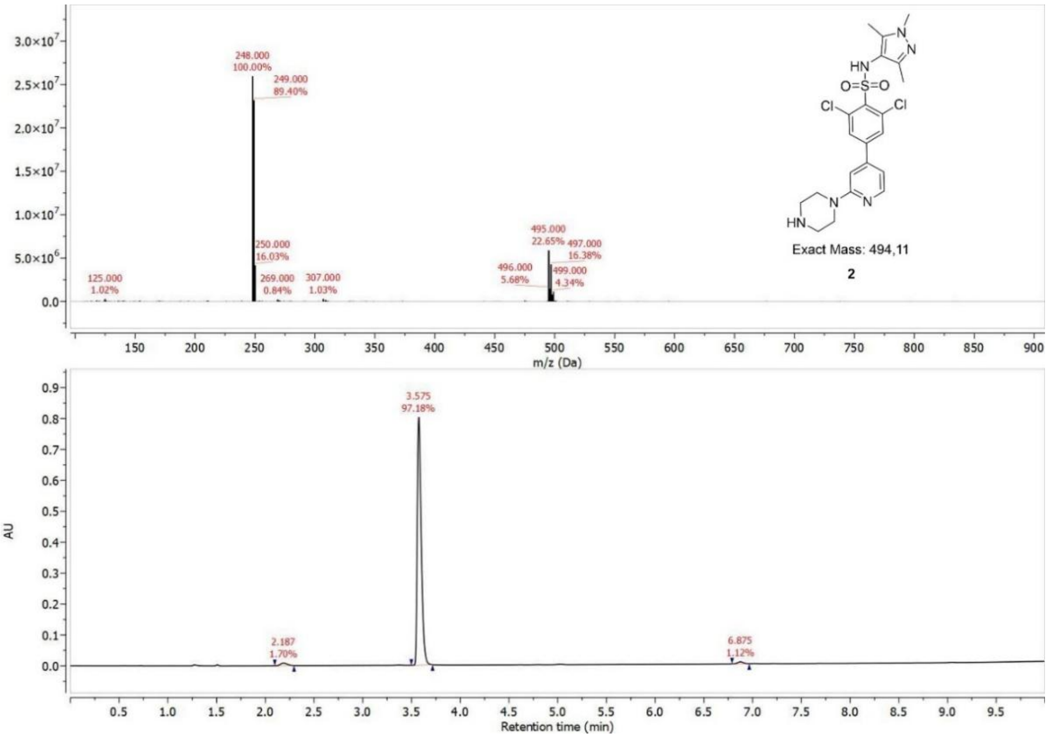

Figure S 10: Mass spectrum and UV chromatogram (254 nm) of compound **2.**

3

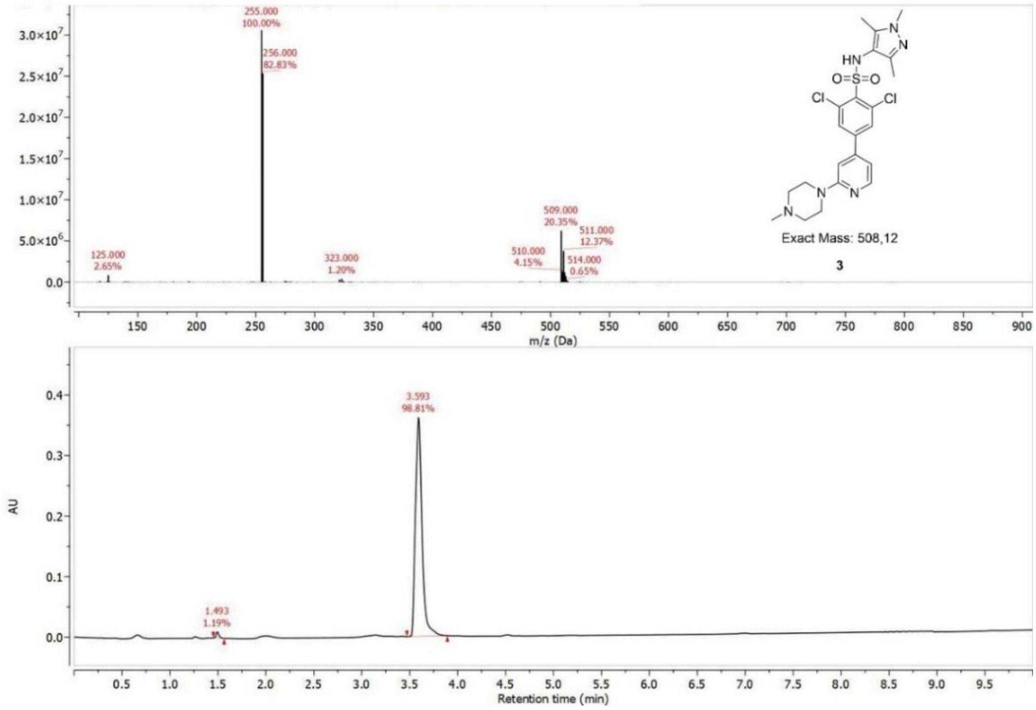

Figure S 11: Mass spectrum and UV chromatogram (254 nm) of compound **3**.

4

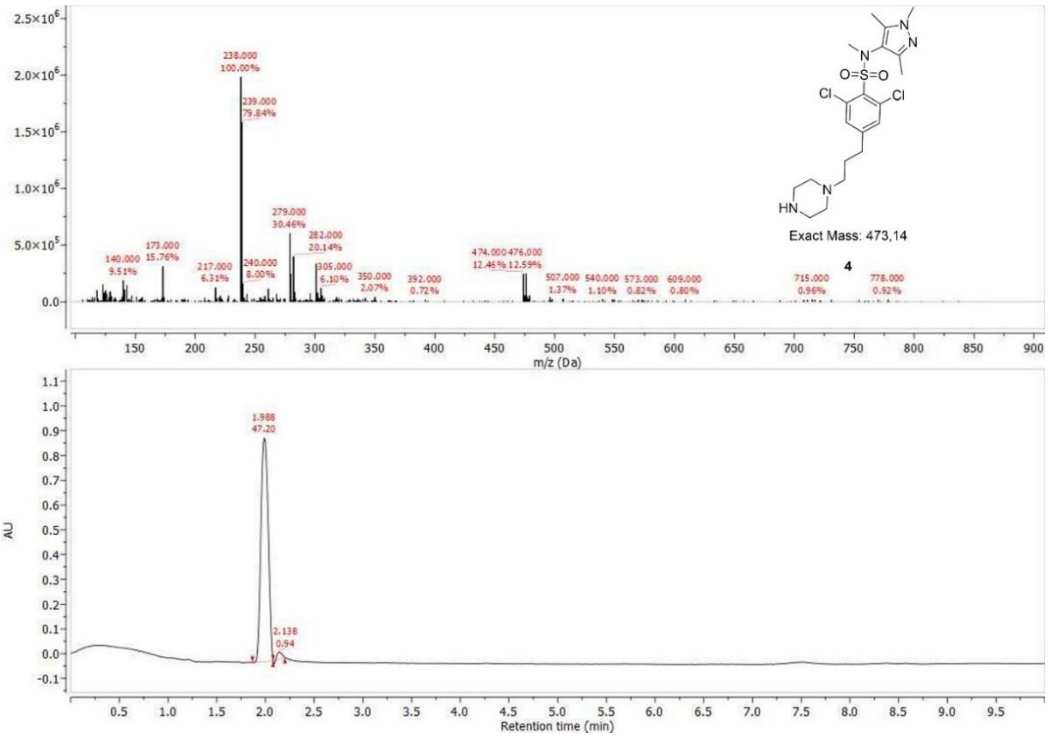

Figure S 12: Mass spectrum and UV chromatogram (210 nm) of compound **4**.

5

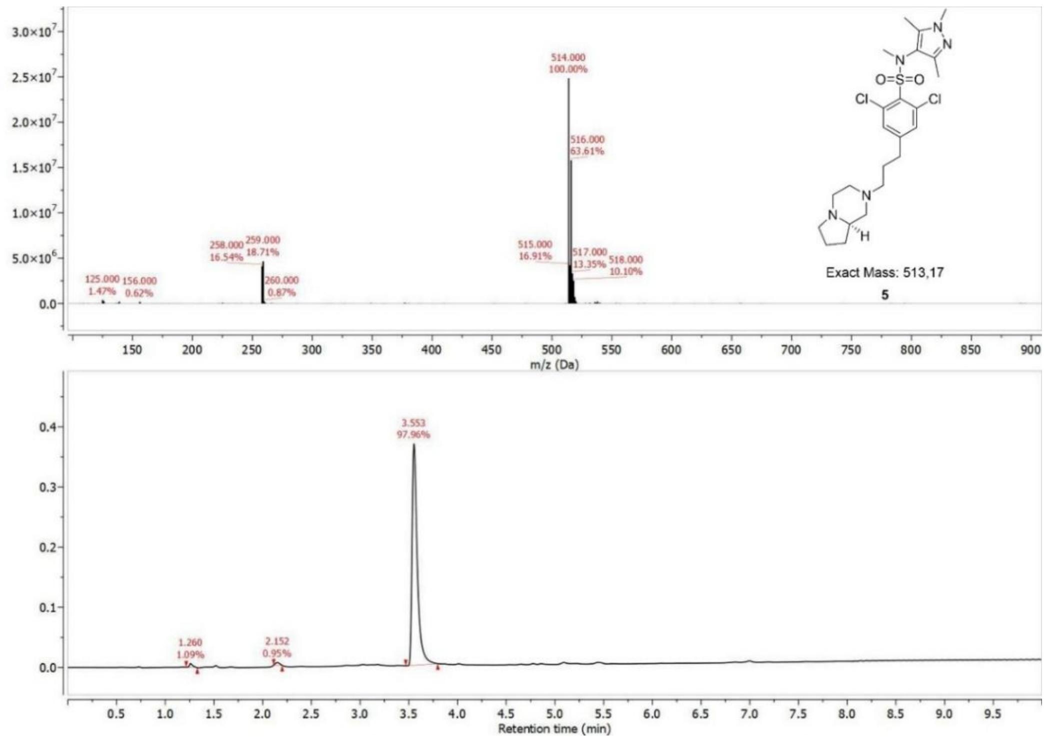

Figure S 13: Mass spectrum and UV chromatogram (254 nm) of compound **5.**

6

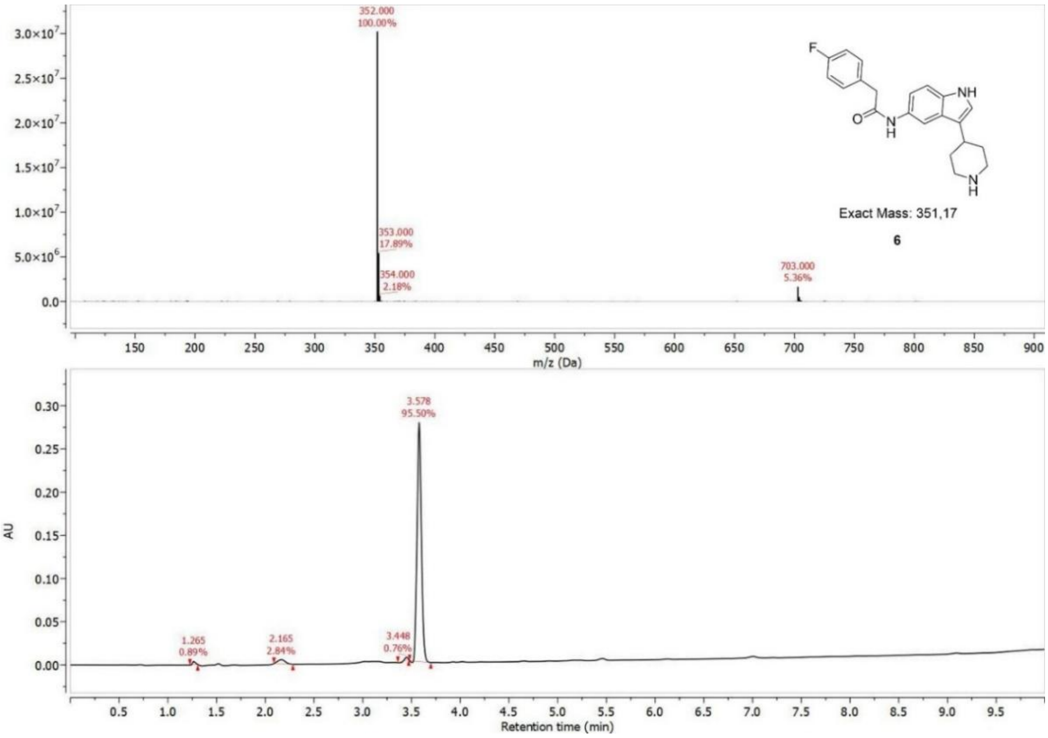

Figure S 14: Mass spectrum and UV chromatogram (254 nm) of compound **6**.
